# Combining two main *NAL1* functional alleles can increase rice yield

**DOI:** 10.3389/fpls.2024.1505679

**Published:** 2024-12-02

**Authors:** Xiang Ouyang, Shuoqi Chang, Xiaoling Ma

**Affiliations:** ^1^ State Key Laboratory of Hybrid Rice, Hunan Hybrid Rice Research Center, Hunan Academy of Agricultural Sciences, Changsha, China; ^2^ Key Laboratory of Cultivation and Protection for Non-Wood Forest Trees of the Ministry of Education, Central South University of Forestry and Technology, Changsha, China

**Keywords:** rice, *NARROW LEAF1*, functional alleles, effective utilization, hybrid vigor, grain yield

## Abstract

*NARROW LEAF1* (*NAL1*) is one of the key genes in regulating photosynthesis and plant architecture. As the antagonistic effects of *NAL1* have concurrent impacts on photosynthesis and yield component traits, how we can effectively utilize the *NAL1* gene to further increase rice yield is not clear. In this study, we used two different main functional *NAL1* alleles, each of which has previously been proven to have specifically advantageous traits, and tested whether the combined *NAL1* alleles have a higher yield than the homozygous alleles. Our results exhibited that the combined *NAL1* alleles had better parent heterosis (BPH) for panicle number and the total filled grain number per plant, and had middle parent heterosis (MPH) for spikelet number per panicle without affecting thousand-grain weight when compared with the homozygous alleles. In consequence, the *NAL1* hybrid plants displayed highly increased grain yield compared with both homozygous parents. The hybrid plants also had better plant architecture and higher canopy photosynthesis. Western blot and proteomics results showed the hybrid plants had a middle abundant NAL1 protein level, and the upregulated proteins were mainly involved in the nucleus and DNA binding process but the downregulated proteins were mainly involved in the oxidation-reduction process, single-organism metabolic process, and fatty acid biosynthetic process. Furthermore, the hybrid vigor effect of *NAL1* was confirmed by substituting the mutual male parent 9311 with 9311-NIL in two super hybrid rice varieties (LYP9 and YLY1). This study demonstrates that we can achieve a higher level of grain production in hybrid rice by using the heterosis of *NAL1*.

## 1 Introduction

Rice (*Oryza sativa* L.) is a staple food crop of the world and it is vital to improve rice yield for global food security. Many effective strategies have been formulated to improve yield production. The Green Revolution, by using the semi-dwarf gene *sd1*, substantially increased the rice yield (Khush, 2001). The efficient use of hybrid vigor in rice displays a yield advantage of 10%–20% over the inbred parental lines (Cheng et al., 2007). Furthermore, improving rice photosynthetic efficiency through the development of C4 photosynthesis or photorespiratory bypasses and screening the critical genomic control loci for the development of ideal plant architecture have also been proposed as effective means to enhance rice yield potential (Zhu et al., 2010; Qian et al., 2016). However, a critical challenge is how to reduce the time from research discovery to true and widespread implementation in agriculture (Bailey-Serres et al., 2019). This might need more elaborate clarification to resolve a complex quantitative trait controlled by multiple factors and the trade-off effects caused by the pleiotropic genes (Ren et al., 2023).

Yield is a complex trait and is multiplicatively determined by the number of panicles per plant, the number of grains per panicle, and grain weight. However, all three components are also complex quantitative traits determined by multiple genes and factors (Xing and Zhang, 2010). Currently, there are a great number of genes that have been found to regulate the three components and affect the grain yield in rice. Though the genes were mapped by focusing on only one trait first, the pleiotropic effects were also subsequently identified and included *GRAIN WIDTH2* (*GW2*) (Song et al., 2007), *PLANT ARCHITECTURE AND YIELD1* (*PAY1*) (Zhao et al., 2015), *IDEAL PLANT ARCHITECTURE1* (*IPA1*) (Jiao et al., 2010), and *SPIKELET NUMBER* (*SPIKE*) (Fujita et al., 2013). *GW2* is the quantitative trait locus (QTL) that negatively controls rice grain width and weight. The phenotype analysis using the near-isogenic lines (NILs) further indicates that *GW2* also negatively affects panicle number but positively increases the grain number per main panicle (Song et al., 2007). *IPA1* has been defined as an ideal plant architecture controlling gene, which can directly bind to the promoter of rice *TEOSINTE BRANCHED1* to suppress tillering. It also can directly and positively regulate the expression of *DENSE AND ERECT PANICLE1* to influence plant height and panicle length (Miura et al., 2010; Lu et al., 2013). *PAY1* is a gain-of-function mutation, causing the trade-off effects of reduced tiller number and increased grain number (Zhao et al., 2015). *SPIKE*, which is an allele of *NAL1*, has pleiotropic effects on total spikelet number per panicle, grain weight, panicle number, and photosynthesis (Fujita et al., 2013; Takai et al., 2013; Ouyang et al., 2022). Given the trade-off effects caused by the pleiotropic genes in yield component traits, plant architecture, and photosynthesis, it is urgent to explore effective ways to apply the genes for higher yield production.

Heterosis mainly refers to a better yield performance of a hybrid offspring compared to its inbred parents and the application of heterosis has achieved great success in improving the yields of major crops worldwide (Hochholdinger and Baldauf, 2018). By using high throughput resequencing technology, many important genomic loci from male and female parents that explained the yield advantage of hybrid rice were found (Huang et al., 2015, 2016; Lin et al., 2020; Lv et al., 2020). *NAL1* has also been identified as one of the key loci of heterosis in two-line hybrid system (Huang et al., 2016). Furthermore, the *NAL1* locus might have an additive effect in regulating grain yield per plant, straw weight per plant, panicle weight, and so on (Lin et al., 2020). These suggest that *NAL1* can be better used in hybrid rice.

Previously, we elucidated two main *NAL1* functional alleles (the full function and partial loss of function alleles), each of which has specifically advantageous traits in regulating the photosynthesis and yield-related traits (Ouyang et al., 2022). In this study, to further explore the potential of *NAL1* in improving rice yield, we tested the combined effect of the fully and partially functional *NAL1* types. Our results show that the *NAL1* hybrid plants had better parent effects for panicle number and the total filled grain number per plant, and a middle parent effect on spikelet number per plant without affecting thousand-grain weight (TGW) when compared to the homozygous parents. As a result, the hybrid plants had better parent heterosis (BPH) for grain yield. The *NAL1* hybrid plants also had improved plant architecture and canopy photosynthesis. Furthermore, to test whether the BPH influence of *NAL1* on biomass production and grain yield is also suitable for two-line hybrid rice, two near-isogenic lines (NILs) of the elite hybrid rice variety Liang-you-pei-9 (LYP9) and Y-liang-you-1 (YLY1) were generated by using 9311-NIL (9311 with the native *NAL1* replaced by the Nipponbare *NAL1*), which resulted in the locus of *NAL1* in LYP9-NIL changing to be homozygous and YLY1-NIL changing to be heterozygous. The field experiment shows that LYP9-NIL had a decreased yield compared with LYP9, but YLY1-NIL had an increased yield compared with YLY1. Our studies provide new insight into effectively using the different *NAL1* alleles to increase rice yield.

## 2 Materials and methods

### 2.1 Plant materials and growth conditions

The *Indica* rice cultivar 9311, which has a partial loss of function *NAL1* allele, and the near-isogenic line (9311-NIL), which is derived from the *Japonica* rice cultivar Nipponbare (Zhang et al., 2014) and has the fully functional *NAL1* allele, were used to generate the *NAL1* hybrid line. The thermo-sensitive genic male sterile line PA64S had the fully functional *NAL1* allele, which was the same as 9311-NIL, and Y58S had the partially functional allele, which was the same as 9311. The near-isogenic sister line of the super hybrid rice variety (LYP9 or YLY1) was generated by crossing the corresponding male sterile line maternal parent (PA64S or Y58S) with 9311-NIL, respectively.

The field experiments were executed in two consecutive summer growth seasons (2022 and 2023) at the experimental station of the China National Hybrid Rice R&D Center (HHRRC) located in Changsha (28.2°N, 113.2°E). Seeds of each line were sown in the seedling nursery and 25-day-old seedlings were transplanted into the field with a density of one plant per 20 cm × 20 cm. The fields were managed according to local agricultural practices for growing rice: 225 kg N ha^–1^, 135 kg P_2_O_5_ ha^–1^, and 250 kg K_2_O ha^–1^.

### 2.2 Leaf type and N content measurement

Flag leaf length, width, and area at the early grain filling stage were measured using a handheld laser leaf area meter (Ci-203, CID, USA). Nine flag leaves from nine plants were measured for each line. For total N content analysis, flag leaves at the early grain filling stage and straws and grains at the maturity stage from the Changsha paddy field were collected separately. Ten replicates, each containing three plants, were measured for each line. The dried samples were milled and analyzed with an NC Analyzer (FlashSmart NC; Thermo Fisher, USA).

### 2.3 Leaf and canopy photosynthesis measurement

The net leaf photosynthetic CO_2_ assimilation rate *(A)* under different photosynthetic photon flux density (PPFD) (*A*–*Q* curve) was measured according to Chang et al. (2020). Measurements were made on the flag leaves at the heading stage between 09:00 am and 16:00 pm on clear days with a portable photosynthesis system (LI-6800, Li-Cor, USA) in the 2023 summer season at Changsha. For each line, five flag leaves from five different plants were used.

Canopy photosynthesis was measured using a canopy photosynthesis and transpiration system (CAPTS-100, MilletHill, China). For each measurement, the chamber (with a size of 100 cm * 100 cm) contained five plants in each line. A total of 25 plants in each line were used. A detailed description of the protocol used for data acquisition and analysis is described by Song and Zhu (2018).

### 2.4 Canopy occupation volume analysis

The plant canopy architecture was analyzed at the grain-filling stage using a plant 3D imaging and modeling system (a-CTP, MilletHill, China). Five plants without any broken leaves in each line were selected. Each plant was photographed at omnidirectional angles individually, and then the 3D plant architecture information was integrated into the point cloud data using Metashape Agisoft software. The point cloud data were reconstructed by the CERS suite software using default values for the analysis parameters.

### 2.5 DIA MS analysis

Proteins of the axillary buds at the tillering stage grown in the paddy field were powdered using liquid nitrogen and extracted with SDT lysis buffer (2% SDS, 100 mM NaCl, and 1/100 volume of DTT). Protein sample concentration was quantified by using a Bradford protein assay kit with BSA. Then each protein sample was hydrolyzed with trypsin (100 ng) and the volume was made up to 100 μL with DB lysis (8 M Urea, 100 mM TEAB, pH 8.5). The samples were subjected to UHPLC-MS/MS analysis. The data analysis and visualization were conducted by Novogene Co., Ltd. (Beijing, China) using the Proteome Discoverer 2.2 (PD 2.2, Thermo Fisher Scientific) platform, Biognosys Spectronaut v. 9.0, and R statistical framework. MS2-based label-free quantification was carried out by analyzing data-independent acquisition (DIA) raw data using Biognosys Spectronaut v.9. Differentially expressed proteins (DEPs) were identified according to a fold change >1.5 and a corrected P-value < 0.05. Functional and pathway analysis of the DEPs was conducted using Gene Ontology (GO) annotation (http://www.geneontology.org/).

### 2.6 Western blot and RT-qPCR

For Western blot analysis, a fragment of a 513 bp coding sequence of *NAL1* was amplified by a primer pair of NAL1-Anti-For1/Rev1 (**Supplementary Table S1**) and was fused into a PET-28a-SuMO vector. The purified fusion protein was injected into rabbits to produce polyclonal antibodies against NAL1. Total proteins were extracted from the axillary bud of *NAL1* plants by using a Plant Total Protein Extraction Kit (Solarbio, Beijing, China). Immunoblots were performed using primary antibodies against NAL1 and ACTIN (Cat# AT0001, Engibody, 1:2000 dilution). After incubating with secondary antibodies HRP-labeled Goat Anti-rabbit IgG (H + L) (Cat# AT0097, Engibody; 1:2000 dilution) and HRP-labeled Goat Anti-mouse IgG (H + L) (AT0098, Engibody; 1:2000 dilution), respectively, the immunoblot signal was visualized using the Immobilon Western HRP substrate (Cat# WBKLS0100, Millipore).

Total RNA was extracted from the axillary bud using TRIzol reagent (Invitrogen). Reverse transcription was performed with the ReverTra Ace qPCR RT Master Mix with gDNA Remover (Toyobo) using 1 μ g total RNA. Quantitative RT-PCR analysis was carried out with a LightCycler 480 engine (Roche) using the LightCycler480 SYBR Green I Master Mix (Roche). The relative quantification method (2^−ΔΔCT^) was used to evaluate the quantitative variation of expression after normalization to *Ubiquitin*. Five replicates of each line were analyzed to produce the mean values for NAL1 expression level. The primers used for RT-qPCR analysis are listed in **Supplementary Table S1**.

### 2.7 Agronomic trait measurement

The plant height, biomass, and yield-related data were collected from plants in each plot, excluding the two marginal columns. The yield-related heterosis contribution rate of *NAL1* was calculated according to Wang et al. (2019). For biomass and yield evaluation of the hybrid rice from LYP9 and YLY1 with the corresponding NIL lines, plants from 2 rows x 4 columns were taken from the inside of the plots to eliminate boundary effects. Four replicates were measured for each line.

## 3 Results

### 3.1 The heterozygous *NAL1* allele had better growth performance than the homozygous alleles

**Figure 1:**
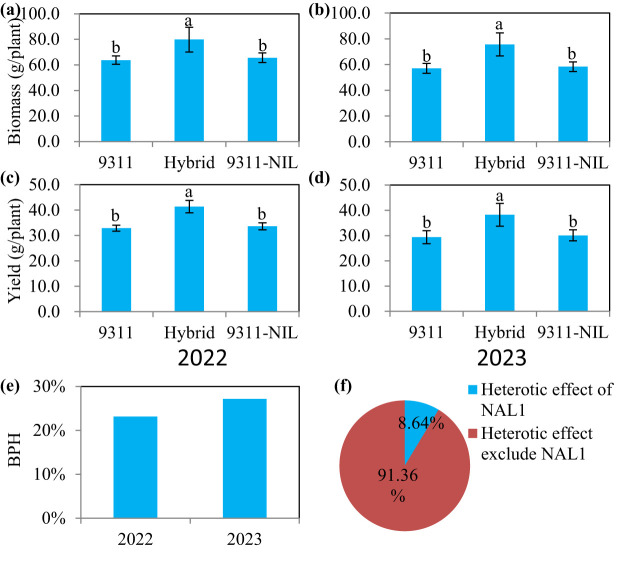
The biomass and yield performance among the three *NAL1* lines grown in two consecutive growth seasons (2022 and 2023) at Changsha. **(A, B)** Biomass. **(C, D)** Yield. The values represent mean ± s.d. (n = 10). Means labeled with different letters indicate significant differences at the 5% level using the Tukey–Kramer test for multiple comparisons. **(E)** The yield-related better-parent heterosis (BPH) of the F1 hybrid. **(F)** The yield-related heterosis contribution rate of *NAL1*.

In a previous study, we elucidated that the fully (9311-NIL) and the partially functional (9311) *NAL1* alleles have different specific advantageous yield-related traits (Ouyang et al., 2022). As the advantageous traits of the two homozygous *NAL1* alleles showed great complementarity, we speculated that heterozygous plants may have a better yield performance than the homozygous plants. To verify this hypothesis, the two elite lines and their crossed offspring were used for further analysis. In the two consecutive years of field trials at Changsha, we found there were no significant difference between 9311 and 9311-NIL in biomass and yield. However, the *NAL1* hybrid plants exhibited significantly higher biomass and yield than both homozygous parents (**Figures 1A–D**). The yield performance of the *NAL1* hybrid plants that exceeded the better parent (named better-parent heterosis, BPH) was up to 27.15% (**Figure 1E**). The solo *NAL1* locus could provide an 8.64% heterosis effect contribution rate (**Figure 1F**). These results suggest the heterozygous *NAL1* variety has improved growth performance and yield production.

**Figure 2:**
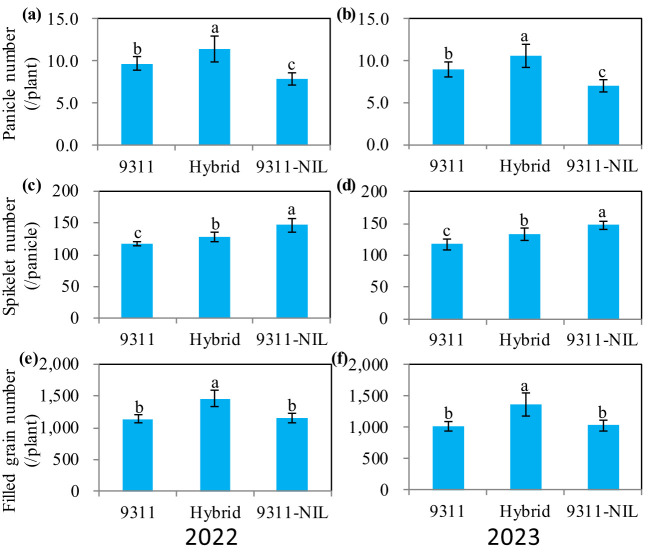
The yield-related traits among three *NAL1* lines grown in two consecutive growth seasons (2022 and 2023) at Changsha. **(A, B)** Panicle number per plant. **(C, D)** Spikelet number per panicle. **(E, F)** Total filled grain number per plant. The values represent mean ± s.d. (n = 10). Means labeled with different letters indicate significant differences at the 5% level using Tukey–Kramer test for multiple comparisons.

We further analyzed the three yield components and found that contrary to TGW, both the panicle number and the spikelet number per panicle were greatly increased in the *NAL1* hybrid plants (**Figure 2**; **Supplementary Figure S1**). When compared with the two homozygous parents, the hybrid plants had significantly higher panicle numbers (**Figures 2A, B**). Even though the number of spikelets per panicle was between that of the two homozygous parents (**Figures 2C, D**), the total filled grain number per plant in the hybrids also showed a significant increase (**Figures 2E, F**). These suggest that the heterozygous *NAL1* allele has BPH for panicle number and the middle-parent heterosis (MPH) for number of spikelets per panicle compared to the homozygous plants.

### 3.2 The heterozygous *NAL1* allele has intermediate leaf type and leaf photosynthesis

**Figure 3:**
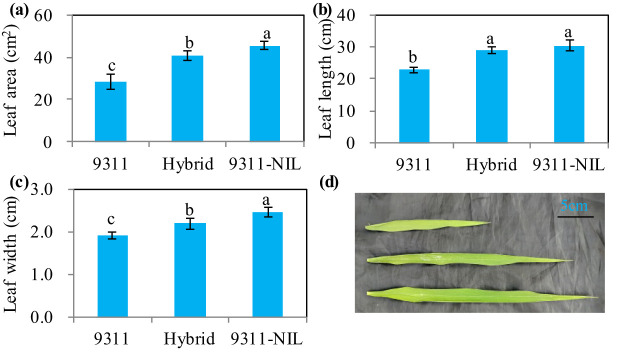
Leaf type among the three *NAL1* lines grown in the field condition at the heading stage. **(A)** Flag leaf area. **(B)** Flag leaf length. **(C)** Flag leaf width. **(D)** Flag leaf morphology. The values represent mean ± s.d. (n = 9). Means labeled with different letters indicate significant differences at the 5% level using the Tukey–Kramer test for multiple comparisons.

**Figure 4:**
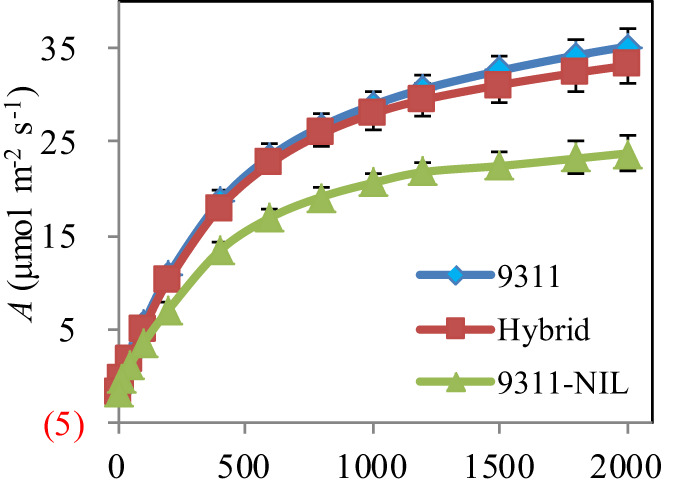
Leaf photosynthesis measurement among the three *NAL1* lines grown in the field. The values represent mean ± s.d. (n = 5).

Leaf type analysis showed that the heterozygous *NAL1* plants had decreased flag leaf length, width and area than 9311-NIL, but the three traits were significantly increased when compared with 9311 (**Figure 3**). Furthermore, *A*-*Q* curve analysis showed that the *NAL1* hybrid plants had similar leaf photosynthetic CO_2_ uptake rate with 9311 under different PPFD, which both were higher than 9311-NIL (**Figure 4**). Although we found no significant differences between the hybrids and two homozygous parents in flag leaf N content, the leaf N accumulation was gradually increased from the 9311, hybrids to 9311-NIL (**Supplementary Figure S2**). These suggest that the heterozygous *NAL1* plants not only have the MPH in leaf type but also have the comparative leaf photosynthetic capacity with parent 9311.

### 3.3 The heterozygous *NAL1* has the optimal canopy photosynthetic efficiency

**Figure 5:**
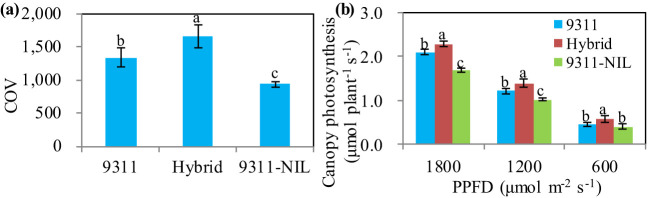
The canopy photosynthesis-related traits among the three *NAL1* lines. **(A)** Canopy occupation volume (COV). **(B)** Canopy photosynthesis. The values represent mean ± s.d. (n = 5). Means labeled with different letters indicate significant differences at the 5% level using the Tukey–Kramer test for multiple comparisons.

Canopy architecture is a major yield determinant of crops because of its effect on light distribution, light interception, and canopy photosynthesis (Zhu et al., 2010; Long et al., 2015). We investigated the canopy architectures of the hybrid plants and the two homozygous parents. The results showed that there were no differences in plant height between the hybrids and the homozygous parents (**Supplementary Figure S3**). The canopy occupation volume (COV), which is a key architectural trait and can reflect the synergistic effect of leaf area index (LAI) and leaf angle on canopy photosynthetic capacity (Liu et al., 2021), showed great variance among three *NAL1* lines. The results showed that 9311 had a significantly higher COV value than 9311-NIL. However, the COV of 9311 was still significantly lower than the hybrids (**Figure 5A**). This suggests that the *NAL1* hybrid plants had greater canopy photosynthetic capacity than 9311 and 9311-NIL. To confirm this, we further investigated the canopy photosynthetic rate of the heterozygous *NAL1* plant and the homozygous parents during the grain-filling stage. The results showed that the hybrid plants had the highest total canopy photosynthetic CO_2_ uptake rate under both high and low light conditions compared with 9311 and 9311-NIL (**Figure 5B**). These results suggest that the heterozygous *NAL1* allele improved canopy architectures and contributed to higher canopy photosynthetic efficiency.

### 3.4 Proteomic responses of the *NAL1* hybrids and parents

A previous study confirmed that NAL1 acts as a serine protease (Li et al., 2023), thus, in order to further illustrate the difference between the parents and hybrid regarding translation levels, we performed a proteomic analysis using axillary buds from 9311, 9311-NIL, and the hybrids. Principal component analysis (PCA) revealed that the replicates of each *NAL1* genotype were clustered into the same group (**Supplementary Figure S4A**). A total of 708 differentially expressed proteins (DEPs) were found to be significantly differentially expressed (corrected P-value <0.05, log2-fold change >1.5) in two compared groups, including 337 DEPs specifically in the hybrid and 9311 compare group (Hy-9311), 295 DEPs specifically in the hybrid and 9311-NIL compare group (Hy-NIL), and 76 DEPs in both groups (**Supplementary Figure S4B**; **Supplementary Data Sheet 1**). There were 177 DEPs upregulated and 236 DEPs downregulated in the Hy-9311 group, and 114 DEPs upregulated and 257 DEPs downregulated in the Hy-NIL group (**Supplementary Data Sheets 2**, **3**). This suggests the total protein expression levels of DEPs in the hybrid plants were downregulated.

**Figure 6:**
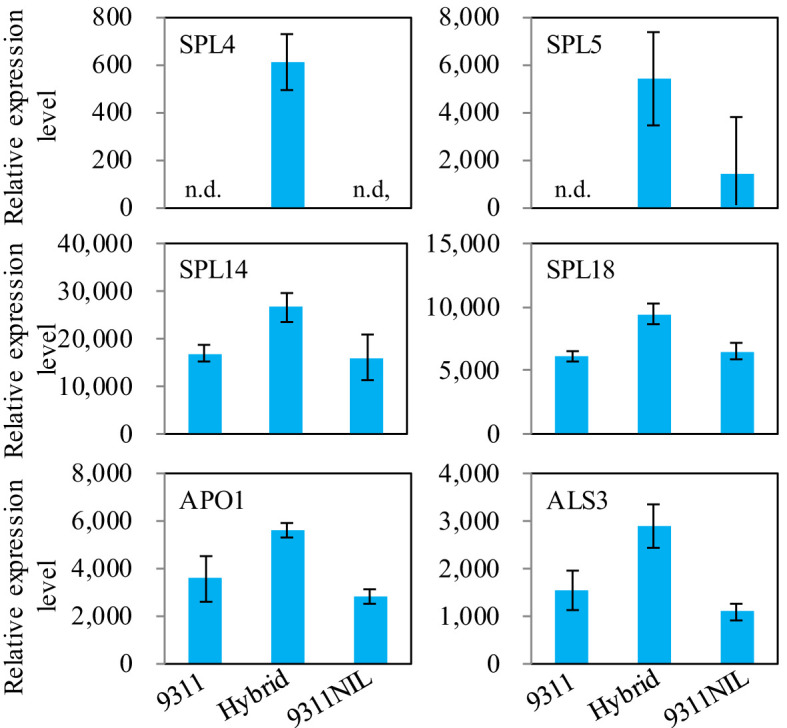
The relative protein levels among the three *NAL1* lines in the proteomic data. The values represent mean ± s.d. (n = 3). n.d., not detectable.

**Figure 7:**
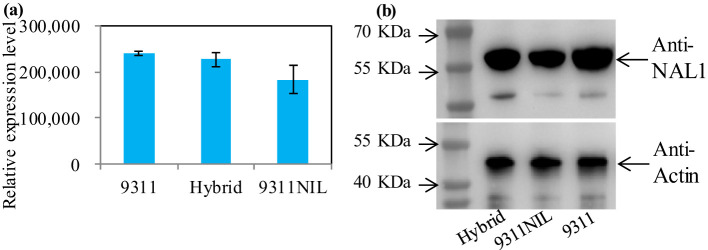
The protein level of *NAL1* among the three *NAL1* lines in young buds. **(A)** The relative NAL1 protein level in the proteomic data. **(B)** NAL1 protein levels analyzed by Western blot. Actin was used as the loading control.

Gene ontology (GO) enrichment analysis showed that most of the downregulated DEPs in the *NAL1* hybrid plants were enriched in oxidation-reduction process, single-organism metabolic process, fatty acid biosynthetic process, and defense response when compared with 9311 or 9311-NIL. In contrast, the upregulated DEPs in the hybrids were mainly involved in DNA binding, nucleosome assembly, and embryo development (**Supplementary Figure S5**). These suggest that the organic components metabolic process might be weakened, but the transcription activity was strengthened in the hybrids. There were several critical proteins were significantly upregulated in the *NAL1* hybrid plants when compared with the parents, such as Squamosa promoter-binding-like proteins (OsSPL4, OsSPL5, OsSPL14, and OsSPL18), OsALS3, and OsAPO1 (**Figure 6**). All of them have been identified to play key roles in regulating the plant architecture and grain yield previously. These suggest that the heterozygous *NAL1* alleles might cooperate with *OsSPLs*, *OsALS3*, and *OsAPO1* to regulate tiller and panicle development. It is interesting that the protein levels of NAL1 gradually decreased from 9311 to the hybrids and to 9311-NIL according to the proteomic data (**Figure 7A**). The Western blot assay further confirmed that 9311 had the highest NAL1 protein level, and 9311-NIL had the lowest protein level by using the anti-body of NAL1 (**Figure 7B**). Furthermore, a similar change pattern for *NAL1* among the three lines was also found in the transcription level (**Supplementary Figure S6**). These results suggest that the *NAL1* hybrids have middle abundant NAL1 protein and transcription levels.

### 3.5 The heterozygous *NAL1* allele has great potential in hybrid rice breeding

**Figure 8:**
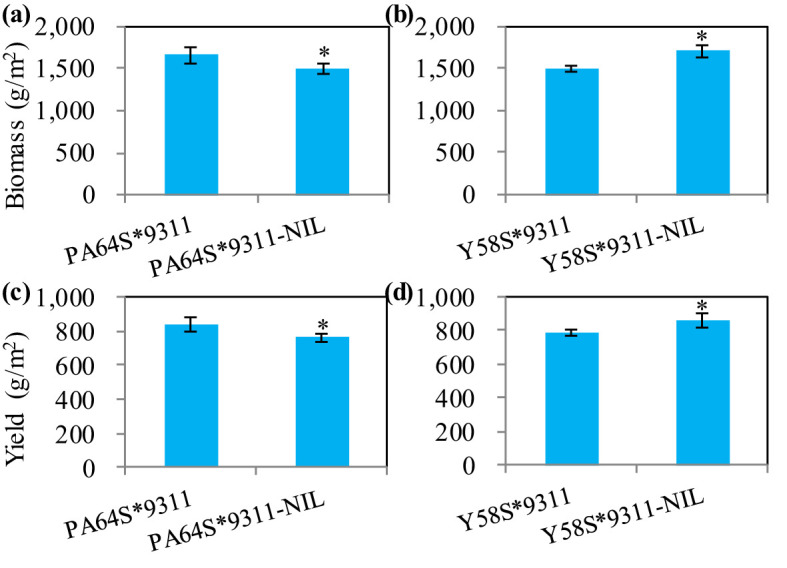
The effects of *NAL1* on biomass and yield in two super hybrid rice varieties, LYP9 and YLY1. **(A, B)** The biomass performance of LYP9 and LYP9-NIL **(A)**, and of YLY1 and YLY1-NIL **(B)**. **(C, D)** The yield performance of LYP9 and LYP9-NIL **(C)**, and of YLY1 and YLY1-NIL **(D)**. LYP9-NIL and YLY1-NIL were generated by hybridizing PA64S or Y58S with 9311-NIL, respectively. The values represent mean ± s.d. (n = 4) with different letters indicating significance using a two-tailed Student’s *t*-test (*p < 0.05).

The above agronomic trait data suggest that *NAL1* would have great value in hybrid rice breeding. To further confirm this, we chose two super hybrid rice varieties, Liang-you-pei-9 (LYP9) and Y-liang-you-1 (YLY1), which shared the same male parent, 9311, but had the different female parents, i.e., PA64S and Y58S, respectively. According to our hypothesis, if we cross 9311-NIL with the female parents PA64S and Y58S separately, the yield of the resulting hybrid lines (LYP9-NIL and YLY1-NIL) will change accordingly. The paddy field experiment results showed a significant decrease in biomass and grain yield in LYP9-NIL when compared with LYP9 (**Figures 8A, C**). The yield reduction was 9.25%. The opposite was also true after the homozygous *NAL1* locus in YLY1 was substituted with heterozygous *NAL1*. When compared with YLY1, YLY1-NIL had an increased grain yield of 10.1% (**Figures 8B, D**). These suggest that the hybrid rice yield can be further increased by using heterozygous *NAL1*.

## 4 Discussion

### 4.1 The hybrid *NAL1* allele plays an over-dominant effect in regulating the source and sink development

Crop yield is a complex trait determined by the balance of photosynthetic assimilates (source) and photosynthate utilization (sink) (Li et al., 2018). A large number of genes have been identified to play important roles in regulating the source and or the sink (Xing and Zhang, 2010; Zuo and Li, 2013; Li et al., 2018; Wang et al., 2018; Ren et al., 2023). *NAL1* has been confirmed to pleiotropically affect plant height, leaf morphology, photosynthesis efficiency, spikelet number, panicle number, and so on. This greatly constrains the application potential of *NAL1* as a result of the trade-off traits related to the source and sink. In this study, we tested an effective method for comprehensively utilizing the advantageous traits of two *NAL1* functional haplotypes. Our results showed the *NAL1* hybrid plants had a larger leaf size than 9311 (**Figure 3**) and had a higher leaf photosynthetic rate than 9311-NIL (**Figure 4**). These results indicate that the combined *NAL1* alleles had synergetic effects on plant architecture and leaf photosynthesis. Furthermore, the canopy photosynthesis capacity of the hybrid plants was also enhanced with the highest panicle number (**Figures 2A, B**), the largest canopy occupation volume (**Figure 5A**), and the highest canopy photosynthetic rate (**Figure 5B**) when compared with 9311 and 9311-NIL. All these results together contribute to a strengthening of the plant source by the combined *NAL1* alleles. Similarly, our results also showed the *NAL1* hybrid plants had the highest panicle number and total filled grain number per plant compared with 9311 and 9311-NIL (**Figures 2A, B, E, F**). This suggests the hybrid *NAL1* allele had over-dominance effects in panicle number and total filled grain number per plant. Moreover, the hybrids had an MPH performance for spikelet number per panicle (**Figures 2C, D**). As a result, the final sink size of the *NAL1* hybrids was increased by the synergistic effect of yield component traits.

### 4.2 The hybrid *NAL1* allele has great potential to improve hybrid rice yield

The role of *NAL1* in rice yield has been identified in many previous studies (Takai et al., 2013; Zhang et al., 2014; Xu et al., 2015; Hirotsu et al., 2017; Ouyang et al., 2022; Li et al., 2023; Zhai et al., 2023; Wang et al., 2024). The ambiguous effect on yield may be due to the concurrent impacts of *NAL1* on source and sink-related traits (Ouyang et al., 2022). As the trade-off effect among yield component traits may be the main reason for a limited rice yield, a balanced or coordinated relationship between the panicle, grain numbers, and grain weight seems to make it easier to produce a high yield. However, it is still important to explore effective ways to apply the genes for higher yield production. One of the effective methods to relieve the trade-off effect might be genome editing of the promoter of the target gene using CRISPR/Cas9 to generate beneficial alleles. This method has the most successful examples in rice (Song et al., 2022) and tomato (Rodriguez-Leal et al., 2017). In this study, we tested another effective way to apply *NAL1* for higher rice production yield. Our result showed the *NAL1* hybrid plants had increased source size and activity which together contribute to significantly higher biomass and yield than both the homozygous parents (**Figures 1A–D**). The calculated heterosis effect contribution rate offered by the solo *NAL1* locus reached 8.64% (**Figure 1F**). Furthermore, when the heterozygous *NAL1* alleles were substituted with homozygous *NAL1* in the super hybrid rice LYP9, the yield reduction was up to 9.25% (**Figure 8C**). In contrast, when homozygous *NAL1* in YLY1 was substituted with the heterozygous alleles, YLY1-NIL had an increased grain yield of 10.1% (**Figure 8D**). These results further confirm the positive over-dominant effect of the *NAL1* locus and the heterozygous *NAL1* allele has great potential to improve rice production yield.

## Data Availability

The original contributions presented in the study are included in the article/**Supplementary Material**. Further inquiries can be directed to the corresponding authors.

## Supplementary material

Supplementary Figure 1 The thousand-grain weight (TGW) among the three *NAL1* lines grown in two consecutive growth seasons in 2022 **(A)** and 2023 **(B)** at Changsha. The values represent mean ± s.d. (n = 10).

Supplementary Figure 2 The flag leaf N status in the three *NAL1* lines. **(A)** Total N content. **(B)** Total N accumulation.

Supplementary Figure 3 The growth performance of the three *NAL1* lines. **(A)** Growth status at the grain-filling stage (Scale bar = 25 cm). **(B)** Plant height. The values represent mean ± s.d. (n = 7).

Supplementary Figure 4 The analysis of proteomic data among the three *NAL1* lines. **(A)** Principal component analysis (PCA). **(B)** Venn diagram of the differentially expressed proteins (DEPs) in the hybrid vs. 9311 and hybrid vs. 9311-NIL comparison groups. The DEGs were identified by using a P-corrected value <0.05, log2 fold change >1.5.

Supplementary Figure 5 GO analysis of the up or downregulated DEPs. **(A, B)** The downregulated DEPs in the hybrid vs. 9311 **(A)** and hybrid vs. 9311-NIL **(B)** comparison groups. **(C, D)** The upregulated DEPs in the hybrid vs. 9311 **(C)** and hybrid vs. 9311-NIL **(D)** comparison groups. BP, Biological processes. CC, cellular components. MF, molecular functions.

Supplementary Figure 6 The transcription level of *NAL1* among the three *NAL1* lines in young buds. The values represent mean ± s.d. (n = 5). Means labeled with different letters indicate significant differences at the 5% level using the Tukey–Kramer test for multiple comparisons.

Supplementary Table 1 The primers used in the recombinant construct for the NAL1 antibody and expression level of *NAL1* in qRT-PCR.

Supplementary Data Sheet Raw data of identified DEPs among the 9311, Hybrid, and 9311-NIL plants in the proteomic analysis.
