# Supplementary material for: Combining two main NAL1 functional alleles can increase rice yield
Source: Front Plant Sci. 2024 Dec 2;15:1505679. doi: 10.3389/fpls.2024.1505679 (PMC11647526; doi:10.3389/fpls.2024.1505679)
Supplement: Supplementary Figure 1 — The thousand-grain weight (TGW) among the three NAL1 lines grown in two consecutive growth seasons in 2022 (A) and 2023 (B) at Changsha. The values represent mean ± s.d. (n = 10). [file Presentation1.pptx]

## Slide 1
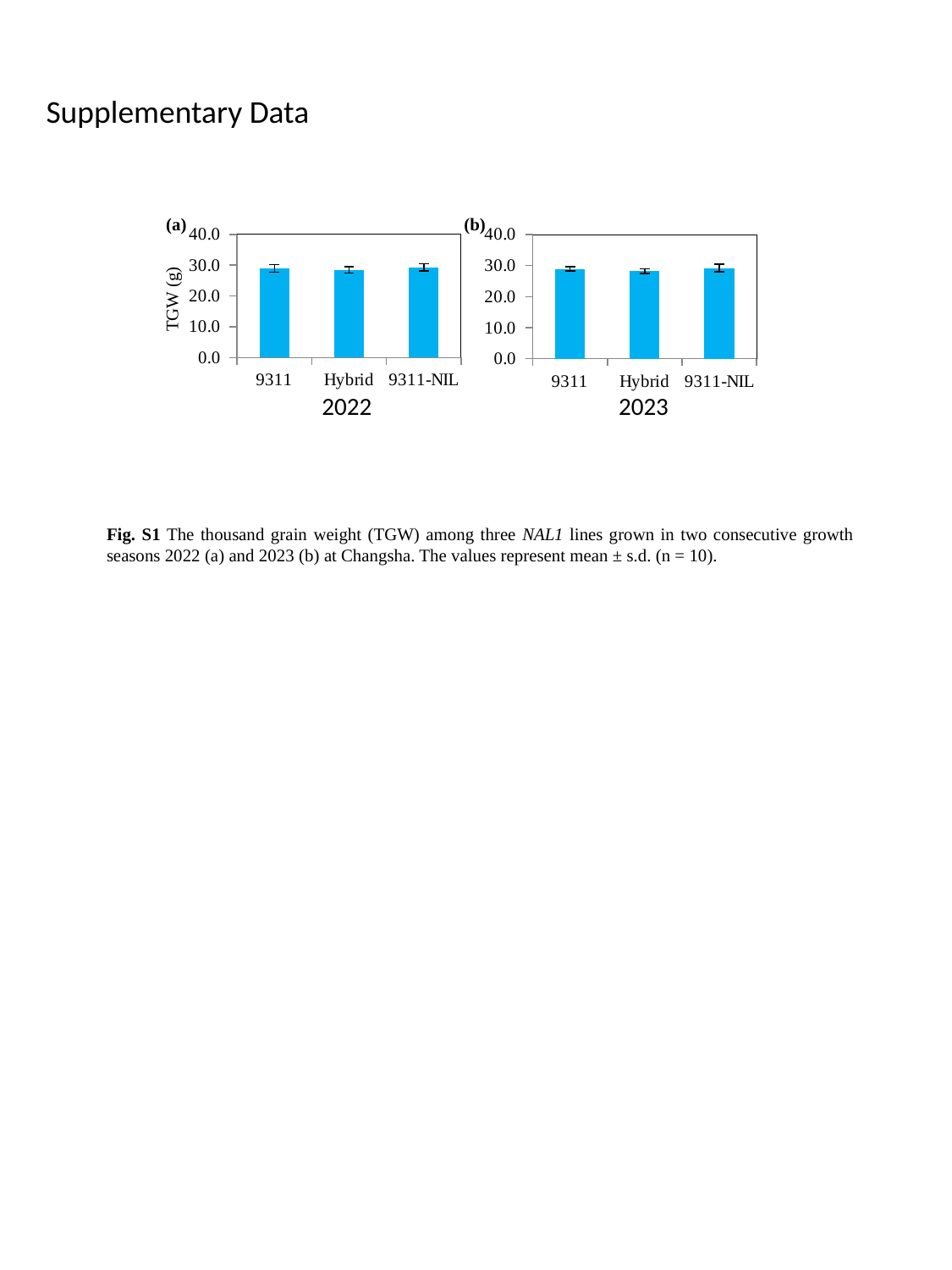

Supplementary Data
(a)
(b)
### Chart
| Category | |
|---|---|
| 9311 | 28.987142857142853 |
| Hybrid | 28.479999999999997 |
| 9311-NIL | 29.294285714285717 |
### Chart
| Category | |
|---|---|
| 9311 | 28.886000000000003 |
| Hybrid | 28.159000000000002 |
| 9311-NIL | 29.176999999999992 |TGW (g)
2023
2022
Fig. S1 The thousand grain weight (TGW) among three NAL1 lines grown in two consecutive growth seasons 2022 (a) and 2023 (b) at Changsha. The values represent mean ± s.d. (n = 10).

## Slide 2
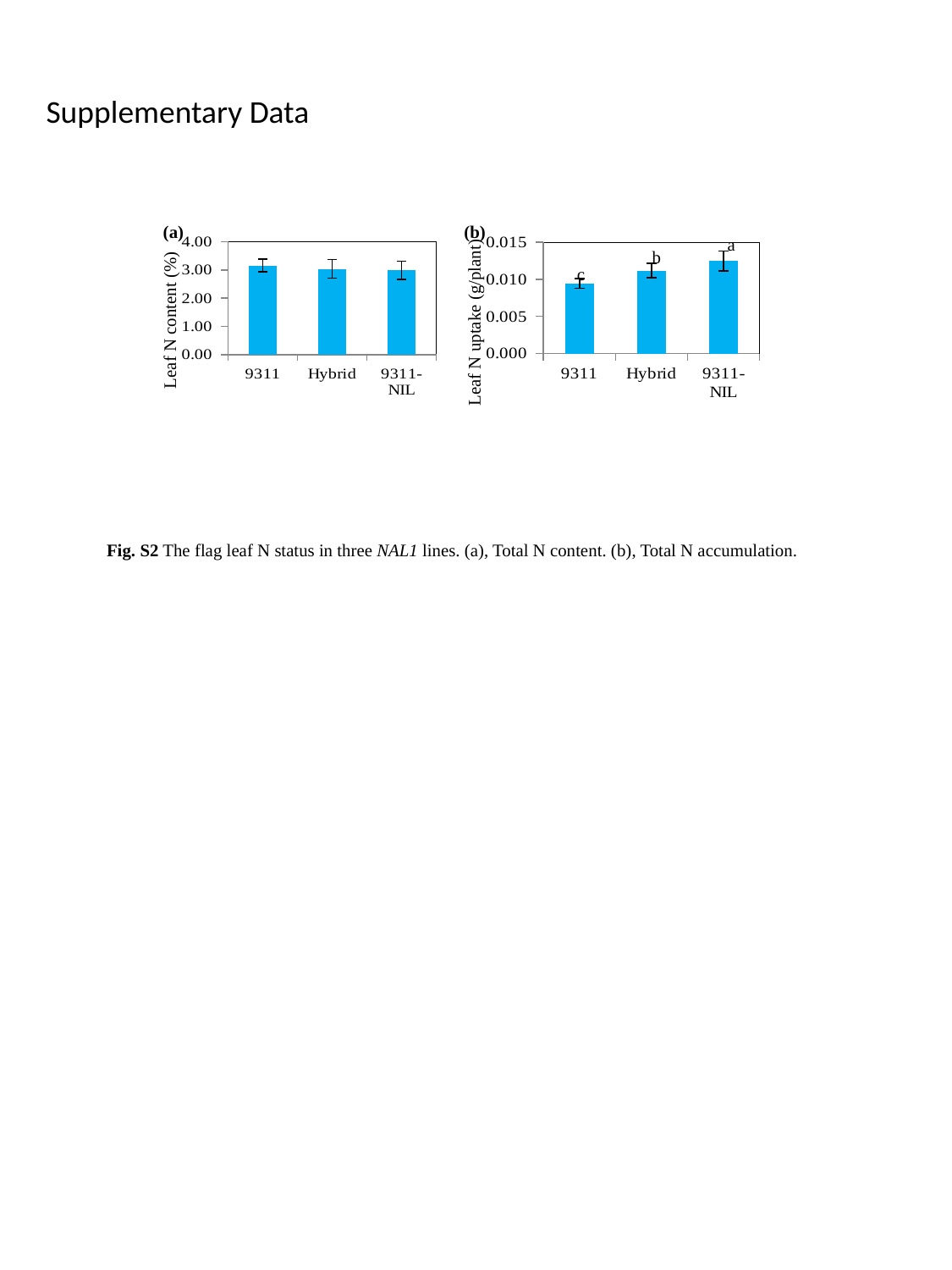

Supplementary Data
(a)
(b)
a
### Chart
| Category | |
|---|---|
| 9311 | 0.009436948625 |
| Hybrid | 0.01115712975 |
| 9311-NIL | 0.012457936374999999 |
### Chart
| Category | |
|---|---|
| 9311 | 3.16 |
| Hybrid | 3.0399999999999996 |
| 9311-NIL | 2.9899999999999998 |b
c
Leaf N content (%)
Leaf N uptake (g/plant)
Fig. S2 The flag leaf N status in three NAL1 lines. (a), Total N content. (b), Total N accumulation.

## Slide 3
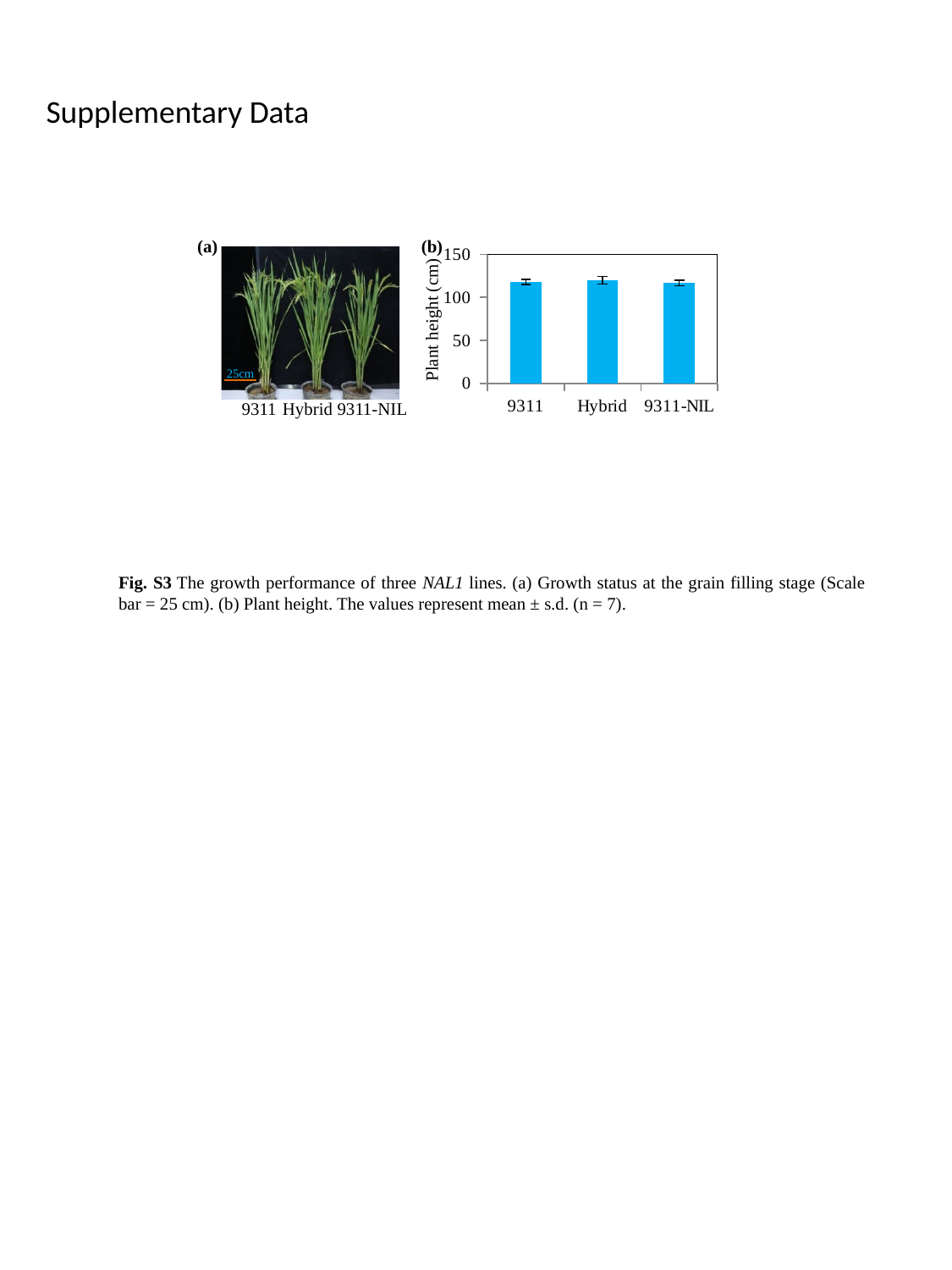

Supplementary Data
(a)
(b)
### Chart
| Category | |
|---|---|
| 9311 | 117.9857142857143 |
| Hybrid | 120.05714285714285 |
| 9311-NIL | 116.88571428571429 |
25cm
9311
Hybrid
9311-NIL
Plant height (cm)
Fig. S3 The growth performance of three NAL1 lines. (a) Growth status at the grain filling stage (Scale bar = 25 cm). (b) Plant height. The values represent mean ± s.d. (n = 7).

## Slide 4
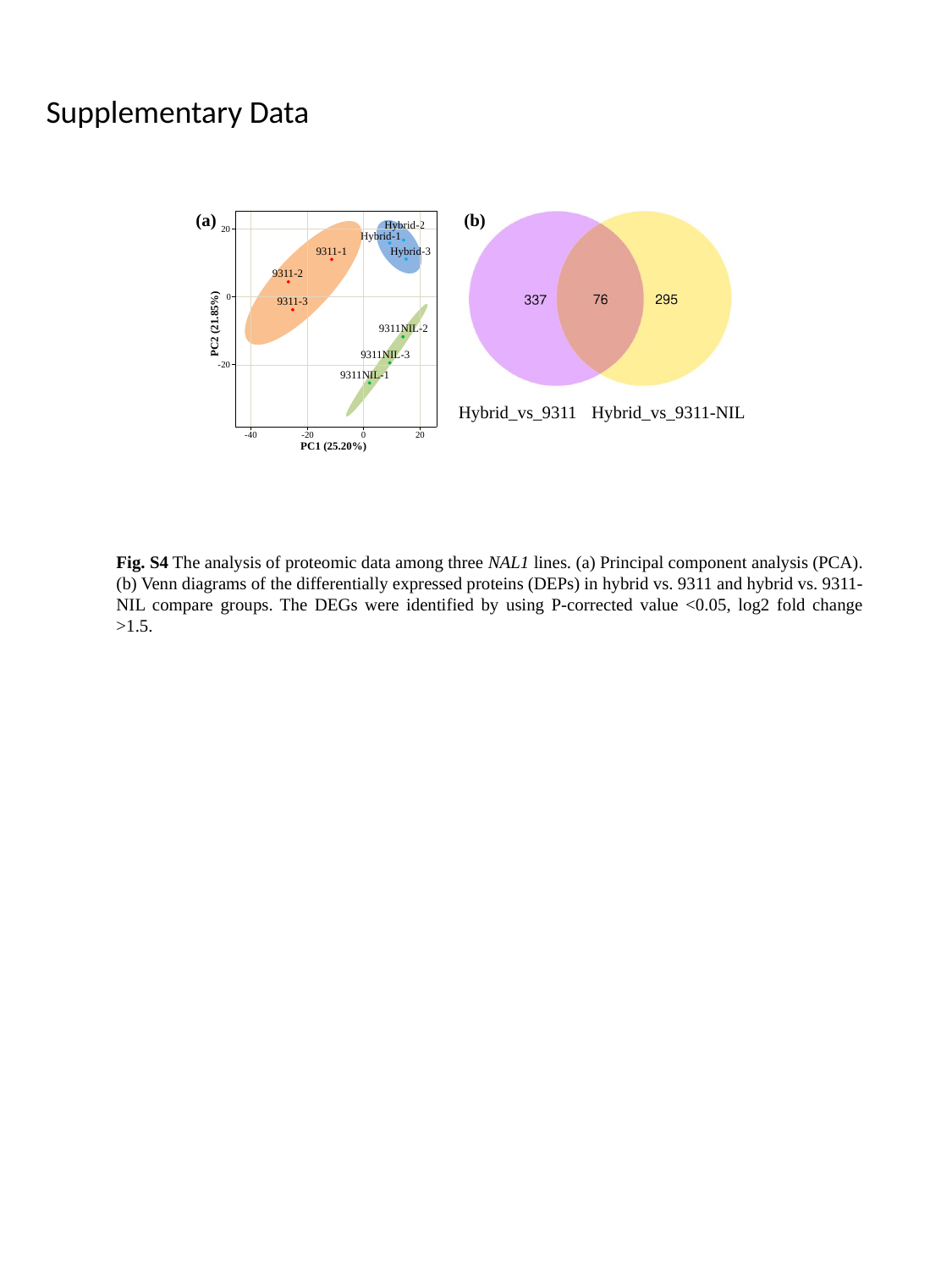

Supplementary Data
B
(a)
(b)
Hybrid_vs_9311
Hybrid_vs_9311-NIL
Fig. S4 The analysis of proteomic data among three NAL1 lines. (a) Principal component analysis (PCA). (b) Venn diagrams of the differentially expressed proteins (DEPs) in hybrid vs. 9311 and hybrid vs. 9311-NIL compare groups. The DEGs were identified by using P-corrected value <0.05, log2 fold change >1.5.

## Slide 5
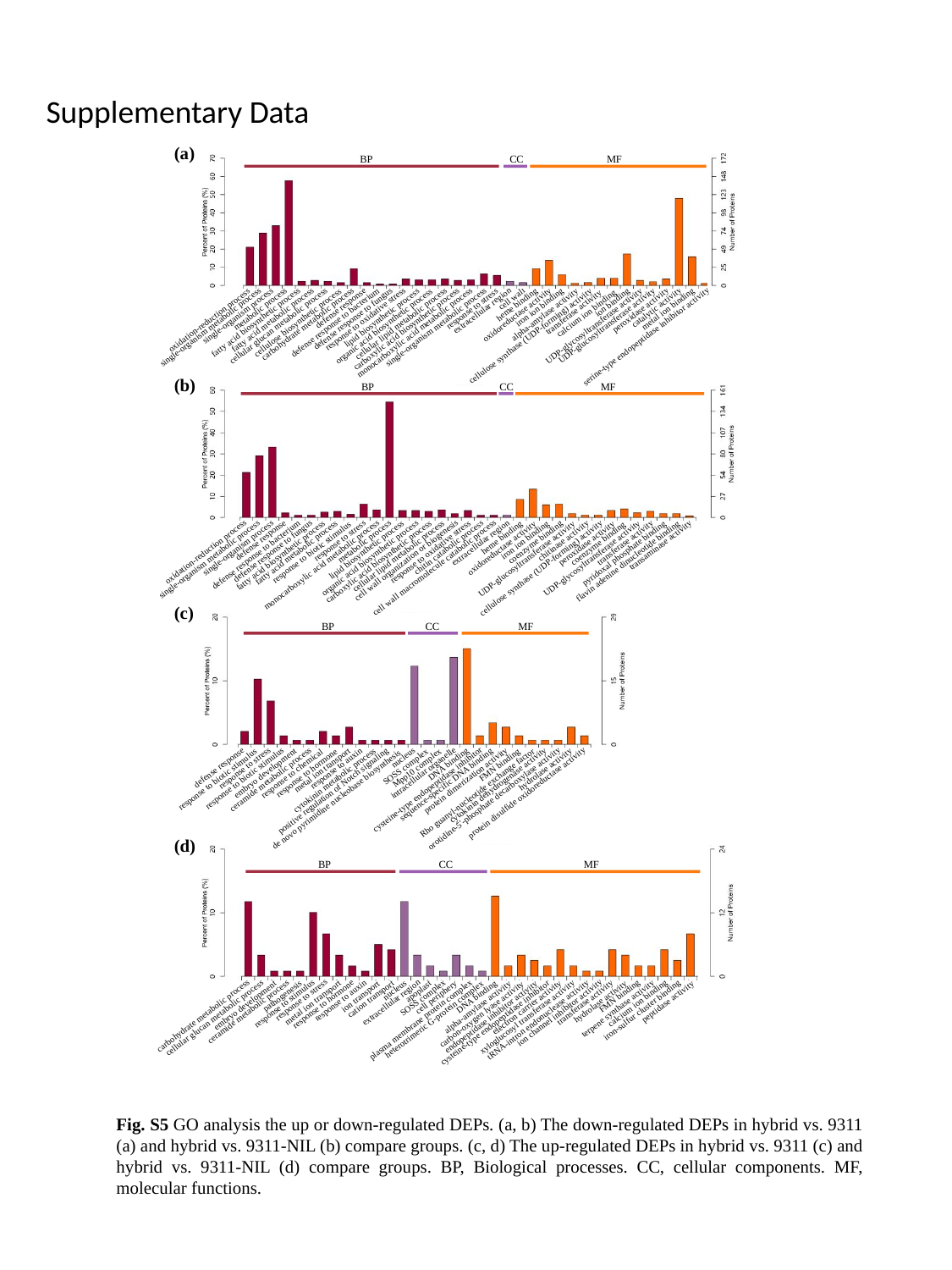

Supplementary Data
(a)
BP
CC
MF
cell wall
ion binding
heme binding
iron ion binding
catalytic activity
defense response
response to stress
metal ion binding
metabolic process
peroxidase activity
extracellular region
transferase activity
calcium ion binding
alpha-amylase activity
oxidoreductase activity
single-organism process
lipid biosynthetic process
defense response to fungus
response to oxidative stress
fatty acid metabolic process
oxidation-reduction process
defense response to bacterium
fatty acid biosynthetic process
cellulose biosynthetic process
cellular lipid metabolic process
carbohydrate metabolic process
UDP-glucosyltransferase activity
cellular glucan metabolic process
UDP-glycosyltransferase activity
organic acid biosynthetic process
single-organism metabolic process
single-organism metabolic process
carboxylic acid biosynthetic process
monocarboxylic acid metabolic process
cellulose synthase (UDP-forming) activity
serine-type endopeptidase inhibitor activity
(b)
BP
CC
MF
heme binding
iron ion binding
defense response
chitinase activity
response to stress
metabolic process
coenzyme binding
transferase activity
extracellular region
peroxidase activity
coenzyme binding
transaminase activity
chitin catabolic process
single-organism process
oxidoreductase activity
lipid biosynthetic process
defense response to fungus
oxidation-reduction process
response to oxidative stress
response to biotic stimulus
fatty acid metabolic process
pyridoxal phosphate binding
defense response to bacterium
fatty acid biosynthetic process
cellular lipid metabolic process
organic acid biosynthetic process
UDP-glycosyltransferase activity
UDP-glucosyltransferase activity
single-organism metabolic process
cell wall organization or biogenesis
flavin adenine dinucleotide binding
carboxylic acid biosynthetic process
monocarboxylic acid metabolic process
cell wall macromolecule catabolic process
cellulose synthase (UDP-forming) activity
(c)
BP
CC
MF
nucleus
DNA binding
FMN binding
SOSS complex
defense response
Mpp10 complex
response to stress
response to auxin
hydrolase activity
metal ion transport
intracellular organelle
response to hormone
embryo development
response to chemical
response to biotic stimulus
response to biotic stimulus
ceramide metabolic process
cytokinin metabolic process
protein dimerization activity
sequence-specific DNA binding
cytokinin dehydrogenase activity
cysteine-type endopeptidase inhibitor
positive regulation of Notch signaling
Rho guanyl-nucleotide exchange factor
protein disulfide oxidoreductase activity
orotidine-5'-phosphate decarboxylase activity
de novo pyrimidine nucleobase biosynthesis
(d)
BP
CC
MF
nucleus
apoplast
FMN binding
pathogenesis
ion transport
DNA binding
cell periphery
SOSS complex
cation transport
response to stress
response to auxin
peptidase activity
hydrolase activity
extracellular region
transferase activity
metal ion transport
calcium ion binding
response to stimulus
response to hormone
embryo development
alpha-amylase activity
electron carrier activity
terpene synthase activity
iron-sulfur cluster binding
ceramide metabolic process
ion channel inhibitor activity
carbon-oxygen lyase activity
carbohydrate metabolic process
endopeptidase inhibitor activity
xyloglucosyl transferase activity
cellular glucan metabolic process
heterotrimeric G-protein complex
plasma membrane protein complex
tRNA-intron endonuclease activity
cysteine-type endopeptidase inhibitor
Fig. S5 GO analysis the up or down-regulated DEPs. (a, b) The down-regulated DEPs in hybrid vs. 9311 (a) and hybrid vs. 9311-NIL (b) compare groups. (c, d) The up-regulated DEPs in hybrid vs. 9311 (c) and hybrid vs. 9311-NIL (d) compare groups. BP, Biological processes. CC, cellular components. MF, molecular functions.

## Slide 6
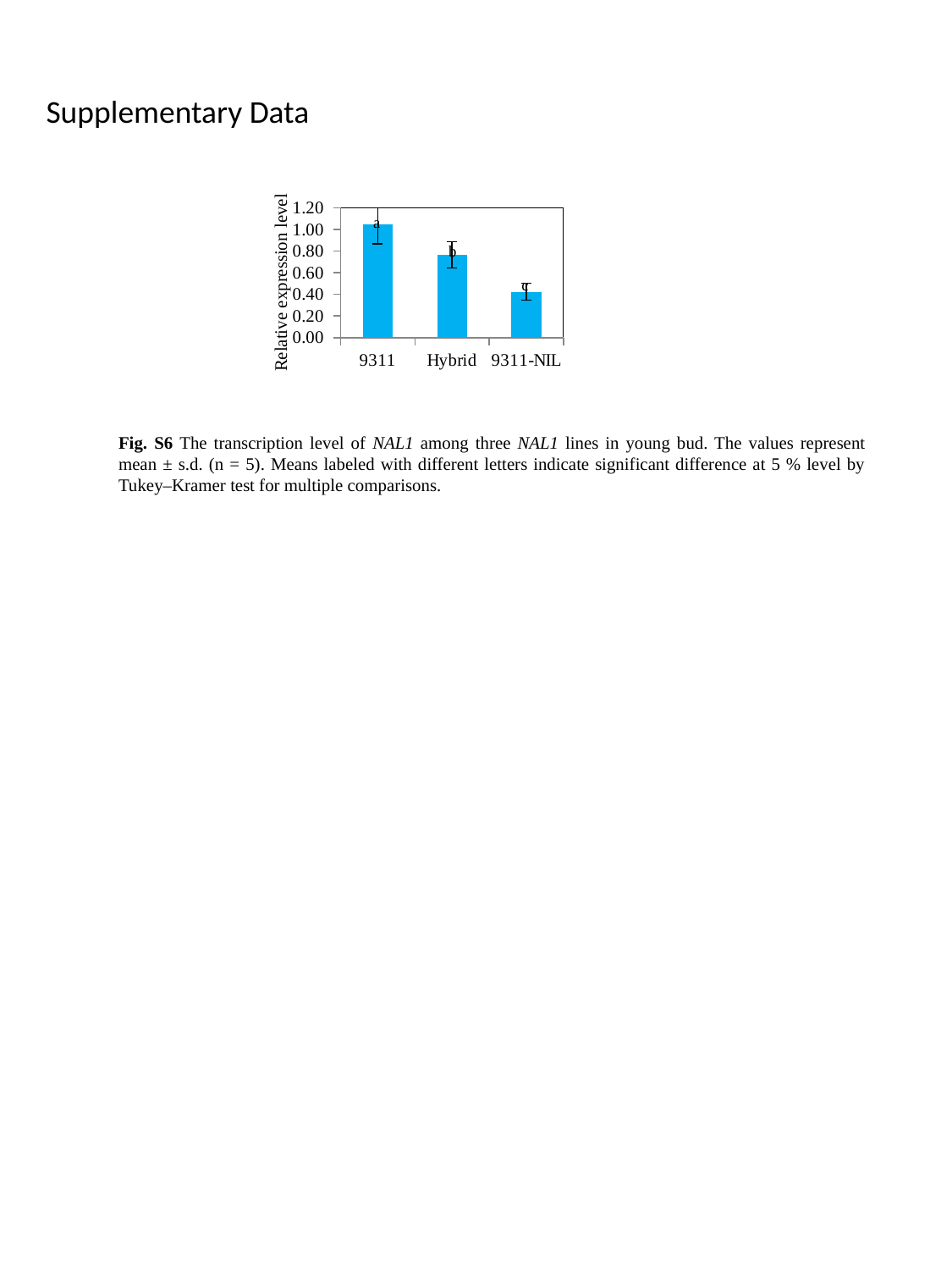

Supplementary Data
### Chart
| Category | |
|---|---|
| 9311 | 1.0458 |
| Hybrid | 0.7654 |
| 9311-NIL | 0.424 |a
b
Relative expression level
c
Fig. S6 The transcription level of NAL1 among three NAL1 lines in young bud. The values represent mean ± s.d. (n = 5). Means labeled with different letters indicate significant difference at 5 % level by Tukey–Kramer test for multiple comparisons.

## Slide 7
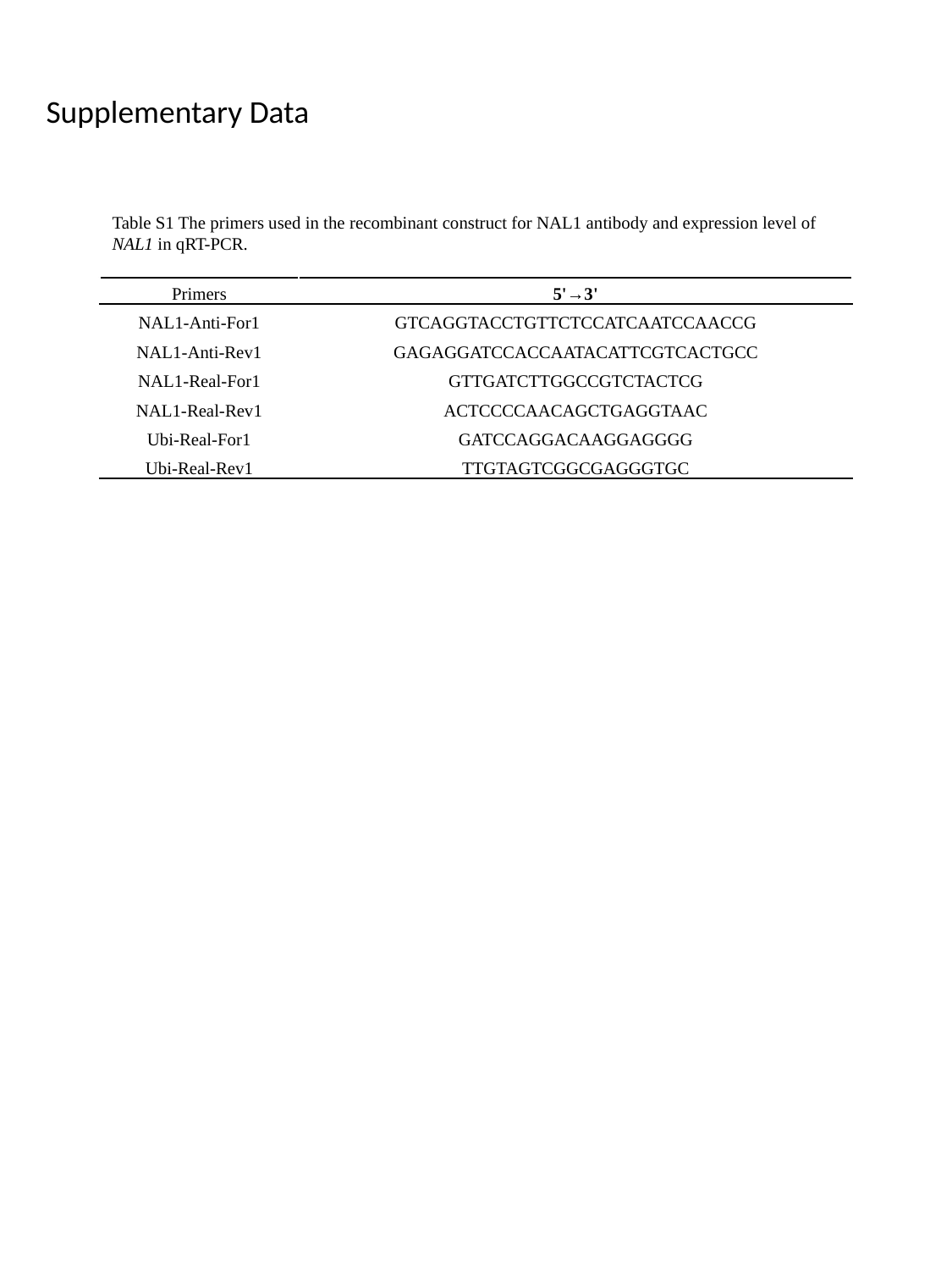

Supplementary Data
Table S1 The primers used in the recombinant construct for NAL1 antibody and expression level of NAL1 in qRT-PCR.
| Primers | 5'→3' |
| --- | --- |
| NAL1-Anti-For1 | GTCAGGTACCTGTTCTCCATCAATCCAACCG |
| NAL1-Anti-Rev1 | GAGAGGATCCACCAATACATTCGTCACTGCC |
| NAL1-Real-For1 | GTTGATCTTGGCCGTCTACTCG |
| NAL1-Real-Rev1 | ACTCCCCAACAGCTGAGGTAAC |
| Ubi-Real-For1 | GATCCAGGACAAGGAGGGG |
| Ubi-Real-Rev1 | TTGTAGTCGGCGAGGGTGC |
